# Aldehyde reduction in a novel pericardial tissue reduces calcification using rabbit intramuscular model

**DOI:** 10.1007/s10856-016-5829-8

**Published:** 2016-12-20

**Authors:** Hao Shang, Steven M. Claessens, Bin Tian, Gregory A. Wright

**Affiliations:** Edwards Lifesciences LLC, One Edwards Way, Irvine, CA 92614 USA

## Abstract

Calcification is a major factor that limits the durability of bioprosthetic valve. A novel bovine pericardial tissue treated with aldehyde capping chemistry and glycerolization was evaluated for its resistance to calcification in comparison with porcine tissues treated with amino oleic acid and bovine pericardial tissue with ethanol rinsing in a rabbit intramuscular model. Tissue discs from the test and control tissues were implanted in rabbits for 60 days. The explanted discs were subject to X-ray imaging, calcium quantification and histology analysis. The test tissue showed 95 and 96 % reduction in calcification in comparison with amino oleic acid treatment and ethanol rinsing treatment, respectively. In addition, the test tissue showed the least inflammatory response as evidenced by a reduced amount of macrophages and giant cells in histology analysis. Furthermore, the aldehyde analysis of the pre-implanted samples showed associated reduction in free aldehyde levels with the test tissue. The reduction in calcification is consistent with previously reported results and is hypothesized to be attributed to the capping of free aldehydes in the test tissue.

## Introduction

Bioprosthetic heart valves have been widely used to replace diseased native valves. Tissue valves, constructed either from porcine aortic valves or from bovine pericardium, have become favored over mechanical valves due to their biocompatibility, hemodynamic superiority and the elimination of the need for life-long anti-coagulant regimens. However, the durability of tissue valves is limited by the progressive structural deterioration primarily due to calcification [1–3].

There are a number of mechanisms underlying tissue calcification including the presence of residual phospholipids and residual free aldehyde functional groups due to glutaraldehyde fixation in tissue preparations, which can be directly correlated to bioprosthetic tissue calcification [4–8]. Therefore, a number of technologies have been developed aimed at mitigating calcification that target these sources using various approaches. Most anti-calcification technologies involve the reduction of phospholipids through the use of various detergents. This approach has proven effective in inhibiting calcification as evidenced by numerous in vivo studies [9–12]. Effective targeting of free aldehydes within the tissue, however, has been more challenging, as most currently available tissue valves rely on the use of glutaraldehyde both during the glutaraldehyde cross-linking process as well as their long-term storage within glutaraldehyde-based preservatives, both sources for the development of free aldehydes. Consequently, the few commercially available technologies targeting free aldehydes have inherent limitations providing long-term protection against calcification via these free aldehydes, as evidenced by the current literature [13, 14].

The limitations inherent within current technologies prompted the development of a novel method of targeting free aldehydes. An aldehyde capping chemistry was developed to covalently cap free aldehydes within bovine pericardial tissue through chemical reduction while preventing tissue from further exposure to glutaraldehyde solutions by glycerolization so that the tissue can be stored dry without a liquid sterilant. The aim of this study was to evaluate the reduction in calcification using this new pericardial tissue in comparison with two commercial tissues containing anti-calcification technologies.

## Materials and methods

### Tissue sample preparation

The test tissue was generated by following a previously described method [15]. In brief, the glutaraldehyde- treated bovine pericardium tissue was treated with an amine and reducing compound to block the residual aldehyde groups. It was followed by glycerolization, which preserved the tissue for dry storage without the need of an aldehyde- containing sterilant. The control samples were harvested from commercially available tissue valves which included: porcine tissue treated with 2-amino oleic acid (AOA) anti-calcification technology (Medtronic, Minneapolis, MN) and bovine pericardial tissue treated with Linx anti-calcification technology (St Jude Medical, St Paul, MN). A 6 mm sterile biopsy punch was used to harvest 4–6 samples per valve leaflet in an aseptic environment. Bovine pericardial tissue fixed in glutaraldehyde with no other treatment was used as positive control.

### Animal implantation

This study was approved in advance by the institutional animal care and use committee at Edwards Lifesciences and was performed in an AAALAC-accredited facility. 6–8 week old New Zealand White rabbits (*n* = 64) were acquired from Western Oregon Rabbitry (Philomath, OR). Rabbits were housed individually or in pairs, depending on their size. The rabbits were allowed to acclimate for at least 6 days after receipt. Before their use in the study, the rabbits’ health was evaluated by either a trained animal care technician or the attending veterinarian. The rabbits were fed a high-fiber commercial diet (LabDiet 5325, Newco Distributors, Ranch Cucamonga, CA) once daily and received water through an automatic watering system ad libitum. The rabbit room was maintained at 16.7–22.2 °C on a 12 : 12-h photoperiod, and humidity was kept between 30 and 70 % with a minimum of 12–15 air changes per hour.

Tissue samples were implanted intramuscularly into rabbits according to the previously published methodology [16]. The intramuscular rabbit model has proven to be a fast and aggressive differentiator of anti-calcification treatment [16, 17]. Each rabbit received one disc from each sample group and the position of the discs was randomized. The rabbits were monitored for pain and discomfort post-operatively. All 64 rabbits survived the implantation and monitoring duration. The discs were retrieved at 60 days after implantation. The discs from two rabbits were explanted with surrounding muscle for histological evaluation. The remainder of the discs were subjected to calcium analysis following host tissue removal. All discs were immediately stored in 10 % buffered formalin upon explant.

### Histological examination

The explanted discs from two randomly selected rabbits were subject to histology examination. Discs were embedded in paraffin and stained with Hematoxylin and eosin (H&E) and von Kossa stain for histological analysis at IDEXX Laboratories (Sacramento, CA). The slides were examined by a blinded investigator under microscope at 25× and 400× magnification for calcification, collagen degradation, host cell infiltration, and inflammatory responses. The number of macrophages and giant cells were counted along the periphery of two sets of tissue samples.

### Tissue calcification analysis

The calcification levels in each disc were qualitatively evaluated by X-ray imaging prior to lyophilization and digested in nitric acid. Calcium levels were quantified by an independent analytical laboratory (Exova, Santa Fe Springs, CA) using ICP-OES (Thermo Fisher Scientific, Waltham, MA). Elemental concentrations were reported as milligram of calcium per gram of lyophilized tissue.

### Aldehyde analysis

The quantification of free aldehyde in the tissue sample was performed as reported previously [18]. A 6 mm tissue disc was punched from each leaflet and was subjected to the aldehyde analysis. A total of ten discs were measured for each tissue sample. The results were expressed as nano-mole aldehyde per disc.

## Results

### Calcification analysis

**Fig. 1:**
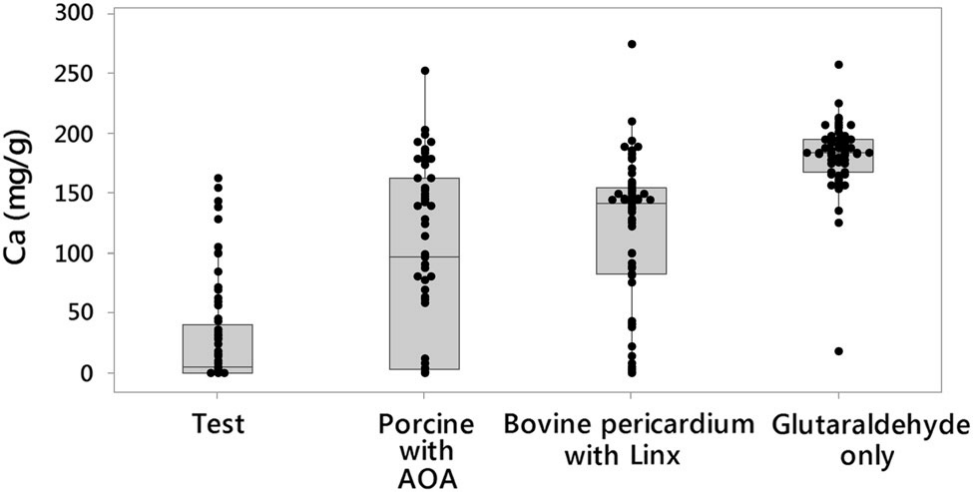
Box and Whisker plot of calcium contents of explanted tissue discs

The quantitative analysis of the calcium are presented in Fig. 1 and Table 1. The large variance of the data, which is typical for animal studies, can be attributed to the variation of the sample and the individual animals. Due to the skewness of the data, Mann-Whitney method was used to compare the median between the groups. The results show the tissues treated with anti-calcification technologies reduced calcification to various degrees over the glutaraldehyde only tissue, with a 23 % reduction for Linx- treated bovine pericardium, 48 % for AOA- treated porcine, and 97 % for the test tissue. In addition, the test tissue showed significantly (*p* < 0.05, Table 2) lower measured calcium content than both Linx- treated tissue and porcine tissue treated with AOA, demonstrating 96 and 95 % reductions, respectively. No significant difference was evident between the AOA and Linx tissue samples.

**Table 1:** Summary of calcium content in the explanted samples (mg/g)

Tissue	*n*	Mean ± StDev	25%, 50%, 75% quantile
Test	62	29.62 ± 43.84	0.12, 5.08, 40.15
Porcine with AOA	62	97.62 ± 74.63	2.66, 96.6, 163
Bovine pericardium with Linx	62	117.26 ± 63.1	82.35, 141, 155
Glutaraldehyde only	62	180.86 ± 29.78	168, 184, 195

**Table 2:** *p*-value of the calcium comparison between the different tissue treatments

	Porcine with AOA	Bovine pericardium with Linx	Glutaraldehyde only
Test	<0.01	<0.01	<0.01
Porcine with AOA	–	0.17	<0.01
Bovine pericardium with Linx	–	–	<0.01

**Fig. 2:**
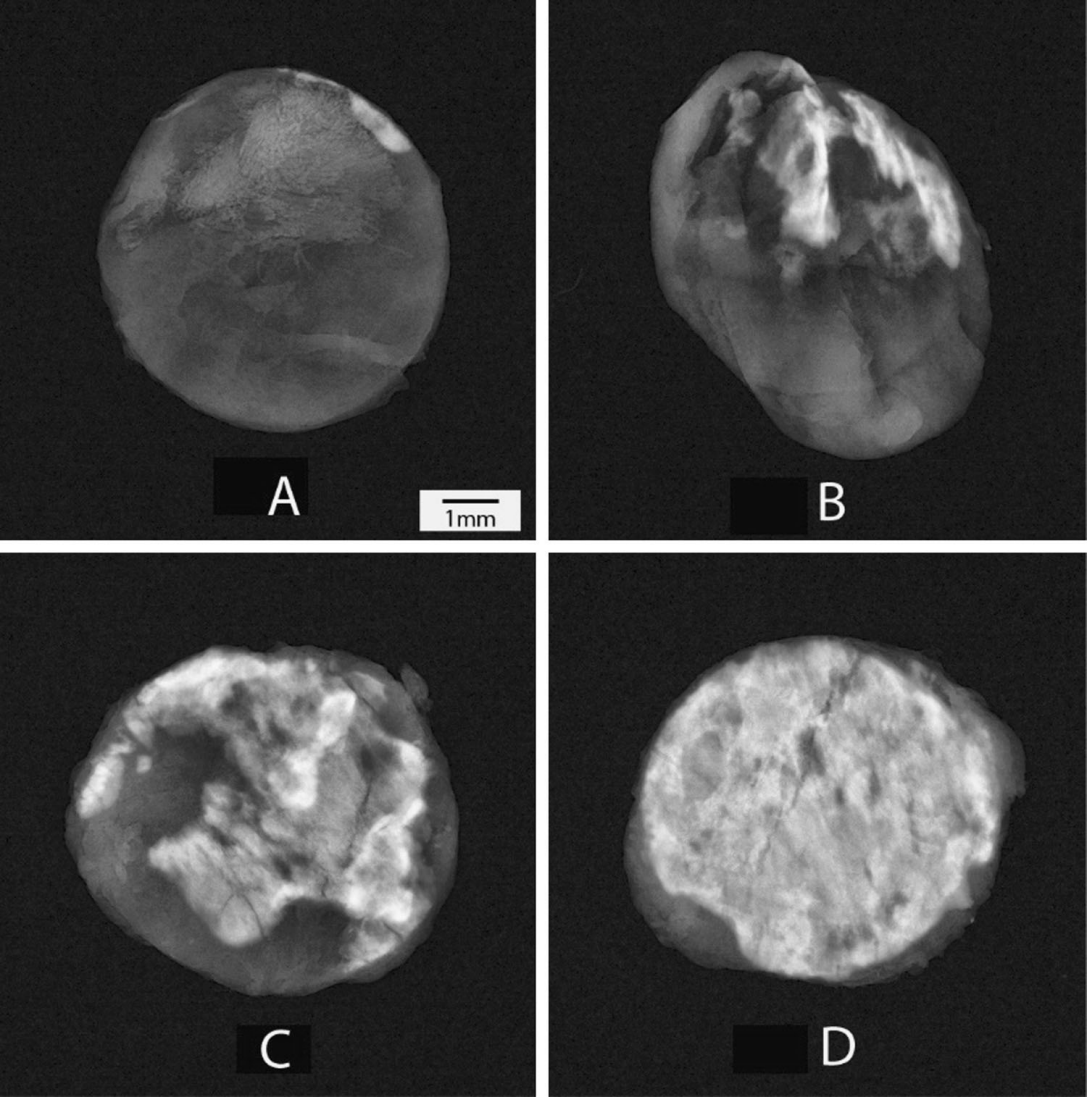
Representative X-ray images of tissue discs after explant of test group (**a**), porcine tissue treated with AOA **(b)**, bovine pericardial tissue treated with Linx **(c)** and bovine pericardial tissue treated with glutaraldehyde only **(d)**. The *white* area represents the calcified nodule. The X-ray images were sorted based on the calcium content and the median calcium images were selected as representative

The calcification nodules are readily visible on the representative X-ray images (Fig. 2). The visual observation shows the lowest calcification for the test tissue, which is consistent with the quantitative analysis.

### Aldehyde analysis

**Fig. 3:**
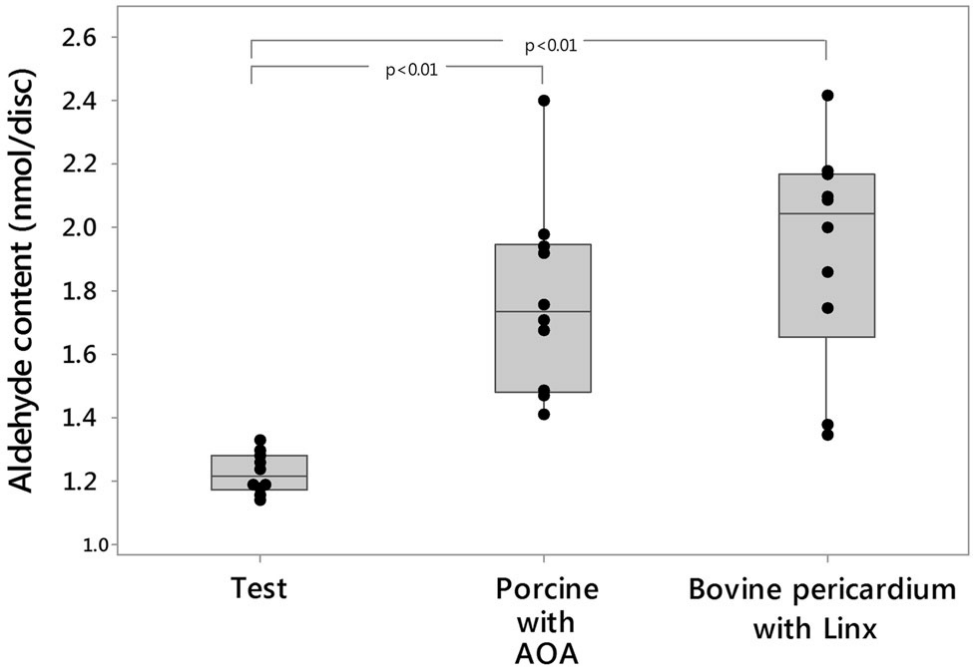
Free aldehyde content of pre-implanted tissue. Ten samples were measured for each group

The free aldehyde content in each tissue was measured (Fig. 3). It is expected that the tissue samples have residual aldehyde due to the glutaraldehyde fixation even after treatment to block the aldehyde groups. The test tissue showed the least amount of free aldehyde among the sample groups. The aldehyde in test tissue was 33.3 and 36.8 % less than AOA- treated and Linx- treated tissue, respectively. In addition, the test group had less variation than the Linx and AOA- treated tissues﻿.

### Histological analysis

**Fig. 4:**
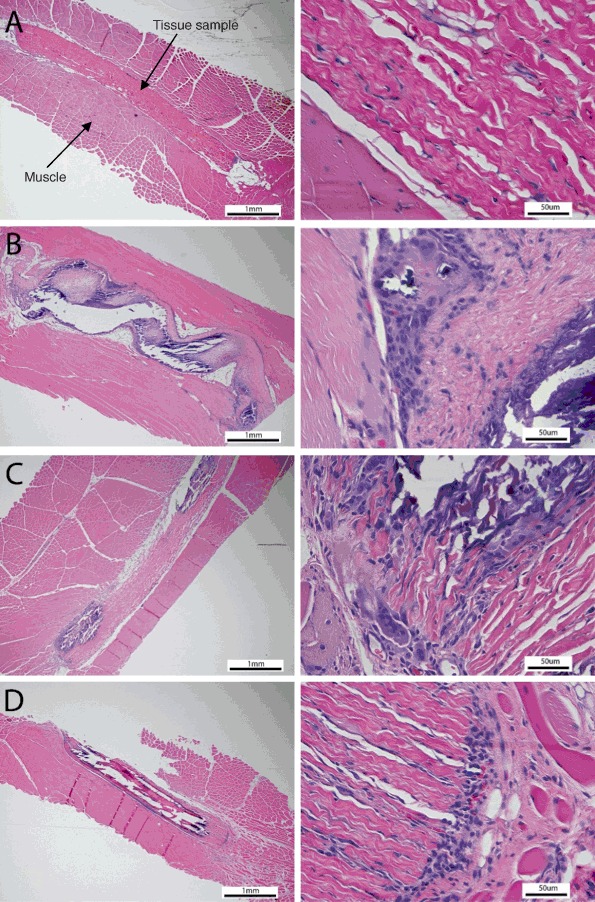
Representative H&E images of test **(a)**, porcine tissue with AOA **(b)**, bovine pericardium with Linx **(c)** and glutaraldehyde only tissue **(d)** tissue after 60 day implant. The *left* images are 25× and the right images are 400×. The *blue* dots are the giant cells and macrophages

**Fig. 5:**
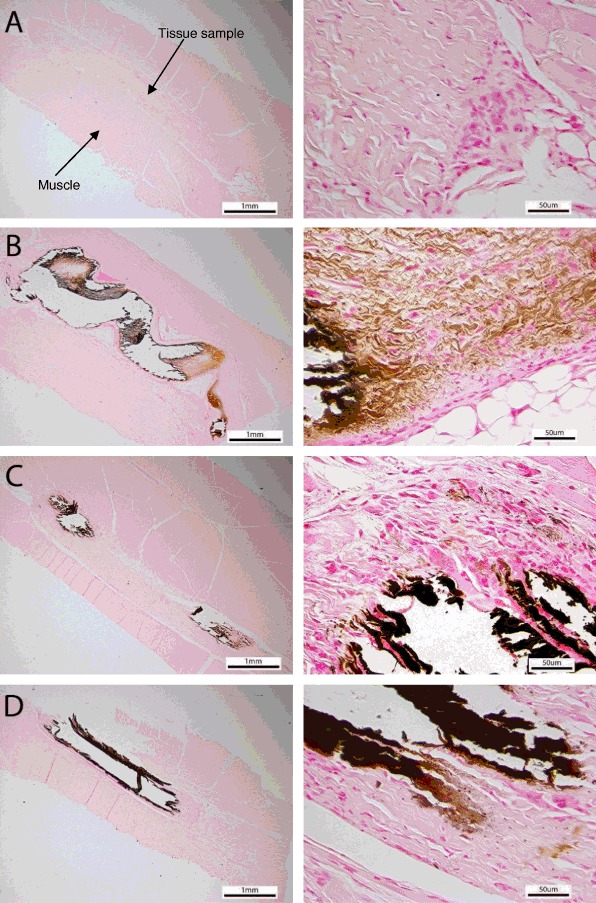
Representative von Kossa images of test **(a)**, porcine tissue with AOA (**b)**, bovine pericardium with Linx **(c)** and glutaraldehyde only tissue **(d)** tissue after 60 day implant. The *left* images are 25× and the *right* images are 400×. The *brown* color is the calcified sites

The overall biocompatibility and calcification of the implants were determined by histological analysis using H&E and von Kossa stains with focus on the inflammatory response, tissue degeneration, and calcification. All the samples showed the typical calcification and foreign body response, such as inflammatory response, but to different degrees. The samples with anti-calcification treatment showed well preserved collagen structure, while the collagen in the glutaraldehyde- only group was largely calcified; this indicates that the anti-calcification treatments are effective in reducing the calcification level and preserving the collagen structure. As shown in the representative histological images from each experimental group, in contrast to the control samples, the test tissue elicited less of an inflammatory response through a visible reduction in macrophage and foreign body reaction giant cell infiltrates (Fig. 4). The counts of macrophages and giant cells from the test and control groups confirmed this observation (Table 3). The AOA- treated tissue showed the most severe chronic inflammatory response among the groups with anti-calcification treatment. No apparent calcification was visible in the representative test tissue explants while tissue calcification was observed in all of the control groups by von Kossa stain (Fig. 5). These findings are consistent with the calcification analysis and X-ray radiography.

**Table 3:** The number of macrophage and giant cell counts for each tissue

	Macrophage			Giant cell		
	Rabbit 1	Rabbit 2	Average	Rabbit 1	Rabbit 2	Average
Test	65	89	77	27	26	27
Porcine with AOA	101	329	215	83	211	147
Bovine pericardium with Linx	124	133	129	74	80	77
Glutaraldehyde only	168	227	198	115	160	138

An average of ten images were captured around the tissue sample and the total number of macrophage and giant cells from the images were counted

## Discussion

Calcification has been the major failure mode for tissue heart valves since their introduction in the 1960s. Improved tissue processing, valve design and application of anti-calcification technologies have contributed to improved durability. However, even with these advancements current expected tissue valve longevity is less than the average life expectancy of patients undergoing aortic valve replacement [19]. Since life expectancy continues to rise in aging populations, there is a critical need to provide a more durable tissue valve, especially one that can mitigate calcification. This newly developed tissue targeting calcification is intended to address this unmet need.

Previously reported results on the safety and efficacy of this new tissue in small animal and large animal studies demonstrate significant improvements in resistance to calcification compared to contemporary valve tissues. The test tissue showed more than 90 % reduction in calcification in comparison with Edwards ThermaFix tissue in a rabbit model similar to the one used in the current study [18]. In both studies, the test tissue consistently showed low levels of calcification (median calcium 6.8 mg/g in the previous study and 5.08 mg/g in the current study). Most notably, Flameng et al. demonstrated statistically significant improvements in hemodynamic performance in valves with the test tissue over standard treated bovine pericardium [15]. While these preclinical studies used Edwards Lifesciences standard bovine pericardial tissue and valves as controls, the current study utilizes other commercial tissue samples as comparators to assess this novel tissue’s efficacy against technologies that employ similar techniques for reducing residual aldehyde groups and phospholipids.

The calcification results confirmed previous study outcomes and showed a significant reduction of calcium content in the test group. The glutaraldehyde only group (positive control) was heavily calcified as expected. The median calcium content of the test tissue group (5.08 mg/g) showed significant reduction in calcium content in comparison with AOA (96 mg/g) and Linx (141 mg/g) treated tissue groups. The test tissue data points were highly concentrated at the low calcium range and had a long positive tail while the other groups were more normally distributed. The long positive tail on the box and whisker may be attributed to the variation of the animal response.

A correlation between aldehyde content and calcification has been established in a previous study, in which pericardial tissue prepared by Edwards was investigated [18]. In the current study, the calcification level also correlates with the aldehyde content reduction across the test tissue and the two controls. The observation suggests that free aldehyde may solicit a host response to trigger calcification, which is consistent with the previous studies [20].

Histological examination of all sample groups demonstrated a typical inflammatory response. The test tissue demonstrated the lowest amount of inflammatory infiltration as evidenced by decreased amounts of giant cells and macrophages in the surrounding host tissue. The collagen structure of the test tissue was well preserved without visible calcification sites, as identified by von Kossa stain. The AOA, Linx and glutaraldehyde-only tissues elicited increased levels of inflammatory response, especially around the calcification sites. These results suggest that valves with this newly developed tissue may offer improved durability, addressing this important unmet need for patients [19].

## Conclusions

The study demonstrates that this novel tissue can reduce calcification by 95 % in comparison with commercially available tissues in the intramuscular rabbit model. The reduction in calcification is consistent with previously reported results, and it is hypothesized that it is attributed to stable capping of free aldehydes and the ability to be stored dry without aldehyde based sterilant. Further studies will be needed to establish the relationship between improved pre-clinical effectiveness and clinical outcomes.

## References

[1] Schoen F, Levy R, Nelson A, Bernhard W, Nashef A, Hawley M (1985). Onset and progression of experimental bioprosthetic heart valve calcification. Lab Invest.

[2] Schoen FJ, Levy RJ (2005). Calcification of tissue heart valve substitutes: progress toward understanding and prevention. Ann Thorac Surg.

[3] Pohle K, Mäffert R, Ropers D, Moshage W, Stilianakis N, Daniel WG (2001). Progression of aortic valve calcification association with coronary atherosclerosis and cardiovascular risk factors. Circulation.

[4] Golomb G, Schoen FJ, Smith M, Linden J, Dixon M, Levy R (1987). The role of glutaraldehyde-induced cross-links in calcification of bovine pericardium used in cardiac valve bioprostheses. Am J Pathol.

[5] Manji RA, Zhu LF, Nijjar NK, Rayner DC, Korbutt GS, Churchill TA (2006). Glutaraldehyde-fixed bioprosthetic heart valve conduits calcify and fail from xenograft rejection. Circulation.

[6] Zilla P, Fullard L, Trescony P, Meinhart J, Bezuidenhout D, Gorlitzer M (1997). Glutaraldehyde detoxification of aortic wall tissue: a promising perspective for emerging bioprosthetic valve concepts. J Heart Valve Dis.

[7] Cunanan CM, Cabiling CM, Dinh TT, Shen S, Tran-Hata P, Rutledge JH (2001). Tissue characterization and calcification potential of commercial bioprosthetic heart valves. Ann Thorac Surg.

[8] Tod TJ, Dove JS (2016). The association of bound aldehyde content with bioprosthetic tissue calcification. J Mater Sci Mater Med.

[9] Vyavahare N, Hirsch D, Lerner E, Baskin JZ, Schoen FJ, Bianco R (1997). Prevention of bioprosthetic heart valve calcification by ethanol preincubation efficacy and mechanisms. Circulation.

[10] Pathak CP, Adams AK, Simpson T, Phillips RE, Moore MA (2004). Treatment of bioprosthetic heart valve tissue with long chain alcohol solution to lower calcification potential. J Biomed Mater Res A.

[11] Jones M, Eidbo EE, Hilbert SL, Ferrans VJ, Clark RE (1989). Anticalcification treatments of bioprosthetic heart valves: in vivo studies in sheep. J Card Surg.

[12] Demer LL (1997). Lipid hypothesis of cardiovascular calcification. Circulation.

[13] Wright G, de la Fuente A (2015). Effectiveness of anti-calcification technologies in a rabbit model. J Heart Valve Dis.

[14] Chen W, Schoen FJ, Levy RJ (1994). Mechanism of efficacy of 2-amino oleic acid for inhibition of calcification of glutaraldehyde-pretreated porcine bioprosthetic heart valves. Circulation.

[15] Flameng W, Hermans H, Verbeken E, Meuris B (2015). A randomized assessment of an advanced tissue preservation technology in the juvenile sheep model. J Thorac Cardiovasc Surg.

[16] Wright GA, Faught JM, Olin JM (2009). Assessing anticalcification treatments in bioprosthetic tissue by using the New Zealand rabbit intramuscular model. Comp Med.

[17] Ozaki S, Herijgers P, Flameng W (2003). Influence of blood contact on the calcification of glutaraldehyde-pretreated porcine aortic valves. Ann Thorac Cardiovasc Surg.

[18] Tod T, Dove J. The association of bound aldehyde content with bioprosthetic tissue calcification. J Mater Sci Mater Med. 2016;27(1):8. 10.1007/s10856-015-5623-z 26610931

[19] Forcillo J, El Hamamsy I, Stevens L-M, Badrudin D, Pellerin M, Perrault LP (2014). The perimount valve in the aortic position: twenty-year experience with patients under 60 years old. Ann Thorac Surg.

[20] Webb CL, Benedict JJ, Schoen FJ, Linden JA, Levy RJ (1988). Inhibition of bioprosthetic heart valve calcification with aminodiphosphonate covalently bound to residual aldehyde groups. Ann Thorac Surg.

